# Complete Genome Sequence of Sphingobacterium sp. Strain ML3W, Isolated from Wings of Myotis lucifugus Infected with White Nose Syndrome

**DOI:** 10.1128/genomeA.01477-14

**Published:** 2015-01-22

**Authors:** Stephen A. Smith, Stephen P. Krasucki, John V. McDowell, Virginia L. Balke

**Affiliations:** Biology & Chemistry Department, Delaware Technical Community College, Newark, Delaware, USA

## Abstract

Sphingobacterium sp. strain ML3W was isolated from the wing of a bat infected with white nose syndrome. We report the complete 5.33-Mb genome sequence of Sphingobacterium sp. strain ML3W, obtained using Pacific Biosciences technology. Being the second complete Sphingobacterium sequence, this will increase knowledge of the genus.

## GENOME ANNOUNCEMENT

Sphingobacterium bacteria have been isolated from diverse niches, including soils and patients with chronic respiratory infections (1). Sphingobacterium sp. strain ML3W is a Gram-negative bacterium isolated from a wing of a little brown bat (Myotis lucifigus) infected with white nose syndrome collected at Tresckow mine, Carbon County, PA. Sterile swabs were used to transfer samples from the bat wings to trypticase-soy agar, and the samples were cultured aerobically at 30°C. 16S rRNA gene sequence analysis placed the isolate in the genus Sphingobacterium. Broth cultures were grown aerobically overnight prior to extraction of genomic DNA using the PureLink Genomic DNA minikit (Life Technologies).

DNA was submitted to the University of Delaware for single-molecule real-time (SMRT) sequencing on the Pacific Biosciences RS II sequencer using 6 SMRT cells with P4-C2 chemistry. Sequencing resulted in 901,752 unfiltered reads and 79,840 filtered reads with a mean read length of 6,539 bases and an *N*_50_ of 9,677 bp. Genome assembly using the Hierarchical Genome Assembly Process (HGAP) (2) yielded one 5,343,049-bp unitig with mean coverage of 75× and a G+C content of 37%. The FASTQ unitig was quality processed using Galaxy (3) (http://www.usegalaxy.org). Galaxy megamerger (4) was used to align 100,000 bp from both ends with a 16-point gap-opening penalty and 4-point extension penalty. The resulting sequence was merged with the 5.3-mb contig and overlapping regions trimmed. The result was a 5,329,011-bp circular chromosome.

The annotation was performed using RAST (5), Prodigal v1.20 Analysis Server (6), and the NCBI Prokaryotic Genome Annotation Pipeline. RAST predicted 4,650 coding genes, Prodigal predicted 4,599, and NCBI predicted 4,093, of which 41 were classified as pseudogenes. Both RAST and NCBI identified 88 tRNA genes and 8 rRNA operons.

Sphingobacterium faecium is the closest neighbor by 16S rRNA sequence comparison, and the closest neighbor identified in RAST is Sphingobacterium spiritivorum. The sequence-based comparison tool in RAST was used to compare the genome of Sphingobacterium sp. ML3W with Sphingobacterium sp. 21, the only other publically available complete Sphingobacterium genome sequence. Very few CDSs show more than 70% homology between the two strains, and the BLAST Dot Plot indicates that there is little similarity in the genome organization. Retron-type RNA-directed DNA polymerase (reverse transcriptase) genes are located in three long regions that are interspersed with genes for phage proteins. Two operons predicted to encode type I restriction-modification systems were found. Subsystem analysis in RAST predicted several genes involved in resistance to toxic compounds, including 25 genes for cobalt-zinc-cadmium resistance, 9 genes for copper homeostasis, 4 genes for arsenic resistance, 1 gene for zinc resistance, and 1 gene for resistance to chromium compounds. Antibiotic resistance genes include 14 genes for β-lactamase, 4 genes for resistance to fluoroquinolones, and 22 genes for multidrug resistance efflux pumps. Similar features were located in Sphingobacterium paucimobilis, which is host to the large PAU phage (7). No genes were predicted for protein secretion systems, and there are more than 60 genes predicted to be TonB-dependent receptors.

### Nucleotide sequence accession number.

The genome sequence has been deposited at NCBI under the GenBank accession no. CP009278.1.
